# HPV Genotyping by Molecular Mapping of Tissue Samples in Vaginal Intraepithelial Neoplasia (VaIN) and Vaginal Squamous Cell Carcinoma (VaSCC)

**DOI:** 10.3390/cancers13133260

**Published:** 2021-06-29

**Authors:** Shitai Zhang, Mayumi Saito, Kaori Okayama, Mitsuaki Okodo, Nozomu Kurose, Jinichi Sakamoto, Toshiyuki Sasagawa

**Affiliations:** 1 Department of Obstetrics and Gynecology, Kanazawa Medical University, Kahoku-gun 920-0293, Japan; zhangshitai@126.com (S.Z.); m-saito@kanazawa-med.ac.jp (M.S.); sakamoto@kanazawa-med.ac.jp (J.S.); 2 Department of Obstetrics and Gynecology, Shengjing Hospital of China Medical University, Shenyang 110004, China; 3 School of Medical Technology, Faculty of Health Science, Gumma Paz University, Takasaki 320-0006, Japan; okayama@paz.ac.jp; 4 Department of Medical Technology, Faculty of Health Sciences, Kyorin University, Mitaka 181-8611, Japan; ohkoudom@ks.kyorin-u.ac.jp; 5 Department of Pathology and Laboratory Medicine, Kanazawa Medical University Hospital, Kahoku-gun 920-0293, Japan; k-nozomu@kanazawa-med.ac.jp

**Keywords:** human papillomavirus, vaginal intraepithelial lesion, uniplex E6/E7 PCR, manual microdissection

## Abstract

**Simple Summary:**

HPV genotypes were determined in 63 vaginal intraepithelial neoplasia (VaIN) and 7 vaginal squamous cell carcinomas (VaSCC). Of these, 37 cases had VaIN alone, and 26 cases had both VaIN and cervical intraepithelial neoplasia (CIN) or condyloma. HPV typing was performed in scraped cells by Genosearch-31 and in the archived tissues by uniplex E6/E7 PCR. In a total of 49 VaIN1, 17 VaIN2/3, and 7 VaSCC tissues, the prevalence of HPV was 91.2% in VaIN and 85.7% in VaSCC. Comparing HPV results in scraped cell and tissue, 46.2% of high-risk (HR) types and 68.1% of any HPV types that had been identified in cell samples were not present in corresponding tissues. HPV types in VaIN and CIN lesions differed in 92.3% of cases with multiple lesions. These results suggest that there are many preclinical HPV infections in the vagina or the cervix, and VaIN and CIN are independently developed. The manual microdissection procedure of tissue revealed one HPV type in one lesion. The vagina appears to be the reservoir for any mucosal HPV type, and HR- or pHR-HPV types are causative agents for vaginal malignancies.

**Abstract:**

HPV genotypes were determined in 63 vaginal intraepithelial neoplasia (VaIN) and 7 vaginal squamous cell carcinomas (VaSCC). Of these, 37 cases had VaIN alone, and 26 cases had both VaIN and cervical intraepithelial neoplasia (CIN) or condyloma. HPV typing was performed in scraped cells by Genosearch-31 (GS-31) and in the archived tissues by uniplex E6/E7 PCR. In a total of 49 VaIN1, 17 VaIN2/3, and 7 VaSCC tissues, the prevalence of HPV was 91.2% in VaIN (VaIN1: 87.8%, VaIN2/3: 100%) and 85.7% in VaSCC. Comparing HPV results in scraped cell and tissue, 46.2% of high-risk (HR) types and 68.1% of any HPV types that had been identified in cell samples were not present in corresponding tissues. HPV types in VaIN and CIN lesions differed in 92.3% (24/26) of cases with multiple lesions. These results suggest that there are many preclinical HPV infections in the vagina or the cervix, and VaIN and CIN are independently developed. The manual microdissection procedure of tissue revealed one HPV type in one lesion. Seventeen HPV types, including high-risk (HR), possible high-risk (pHR), and low-risk (LR), were identified in 43 VaIN1 lesions. In higher grade lesions, six HR (HPV16, 18, 51, 52, 56, 58), one pHR (HPV66), and one LR (HPV42) HPV types were identified in 17 VaIN2/3, and six HPV types, including HPV16, 45, 58, and 68 (HR), and HPV53 and 67 (pHR), were detected in each case of VaSCC. The vagina appears to be the reservoir for any mucosal HPV type, and HR- or pHR-HPV types are causative agents for vaginal malignancies.

## 1. Introduction

Human papillomavirus (HPV) has been proven to be the causative agent of cervical cancer and its precancerous lesions, this virus belongs to the Papillomaviridae family, which is a small, non-enveloped type, double-strand DNA (dsDNA) virus group [1]. To date, more than 200 types had been identified on the basis of DNA sequence data [2,3], and about 50 HPV types have been identified in the mucosal epithelium of the uterine cervix, the vagina, and the vulva [4]. A group of approximately one dozen forms of HPV comprise the main etiologic factors for the development of neoplasms of the lower genital tract of women. It is estimated that 70–80% of all women are infected with cervical HPV at some point during their lifetime, and 90% of these infections can be eliminated within a few years [5,6]. However, 10% of women fail to clear the virus, and long-term persistent infection induces cervical cancer [6,7]. On the basis of their association with premalignant and malignant lesions, 13 HPV types (HPV16, 18, 31, 33, 35, 39, 45, 51, 52, 56, 58, 59, and 68) have been defined as high-risk (HR), although HPV68 is categorized as a probable high-risk type [8]. An additional nine HPV types (HPV26, 30, 34, 53, 66, 67, 70, 73, and 82), which belong to alpha-5, 6, 7, 9 and 11 genera, are thought to be possible high-risk HPV (pHR) types because these types are identified in cancer tissue [9]. HR-HPV encodes two oncoproteins, E6 and E7, which are continuously transcribed in premalignant and malignant tissues. The E7 oncoprotein binds to the tumor-suppressive retinoblastoma protein (pRb), and this interaction facilitates host cell proliferation by inhibition of the function of pRb. The E6 oncoprotein also down-regulates p53, which functions as a gatekeeper protein that regulates cell proliferation and induces apoptosis of the cells damaged by UV-stimualtion [10]. The E6 oncoprotein also inhibits a group of proteins having the PDZ-motiff [11,12]. Thus, E6 and E7 act synergistically to drive host cell proliferation without regulation, and transform these cells into cancer cells. However, it is thought that such actions are generally not present in low-risk (LR) HPV types.

Although the causal relationship between HPV infection and cervical SIL and cancer development has been confirmed by many studies, the role of HPV in the vagina is not well understood. One reason for this is that vaginal squamous cell carcinoma is a rare disease, and HPV-positivity in the cancer (60–70%) is less than that in cervical squamous cell cancer (more than 95%) [13,14]. Another report showed that 93.6% of vaginal intraepithelial lesions (VaINs) are positive for HPV [15]. We have also reported that various HPV types are identified in the vaginal lesions, and HPV is positive in 84% of VaIN1 cases and 100% of VaIN2/3 cases [16]. Highly sensitive HPV testing has shown that most VaIN cases are positive for HPV, although a few cases of VaIN1 have been shown to be negative for HPV [13,16]. During the carcinogenic process after HPV infection, in addition to the HPV genotype, the infection site may also be important. Many studies suggest that infection in certain cell populations located in the squamous-columnar junction (SCJ) of the cervix is a crucial step for cancer development [17]. We have previously reported that multiple HPV types can be frequently identified in the cervical cell samples.^16^ However, it has been reported that one HPV type is found in one lesion, including cervical cancer and high-grade SILs (CIN2 or CIN3) [18]. This suggests that scraped cell samples from the cervix are likely to contain different cell populations from various lesions, including the vaginal lesions. Therefore, cell samples are not suitable for the determination of the HPV type responsible for the target lesion. Recently, it was found that a combination of laser capture microdissection (LCM) and highly sensitive HPV testing may be the most effective test to clarify the relationship between a certain HPV type and the related lesion [19]. Alternatively, Snow AN et al. [20] also introduced manual microdissection with small FFPE (formalin fixed and paraffin embedded) tissue specimens as a simple and cost-effective method to perform analysis of certain genes. We recently established a highly sensitive HPV-PCR method, termed uniplex E6/E7 PCR, which is able to identify the E6 or E7 genes of 39 HPV types, including all HR and pHR types, and some LR HPV types [21]. 

The aim of this study was to determine the HPV genotype responsible for VaIN using molecular mapping of target lesions in FFPE tissue specimens and the uniplex E6/E7 HPV-PCR test. We attempted to clarify the clinical–pathological classification of the HPV type in VaIN. Primary HPV test screening has been recently launched in some Western countries [22], and VaIN is occasionally found in cervical cancer screening. Therefore, this kind of study may be important for clinical management for VaIN after screening, and for knowledge about the mechanism of vaginal cancer induced by HPV infection. This is the first study to determine HPV types by molecular mapping of VaIN lesions, and may elucidate the HPV types responsible for low-grade lesion (VaIN1), high-grade lesion (VaIN2/3), and VaSCC.

## 2. Materials and Methods

### 2.1. Selection of Subjects and Specimens

**Figure 1:**
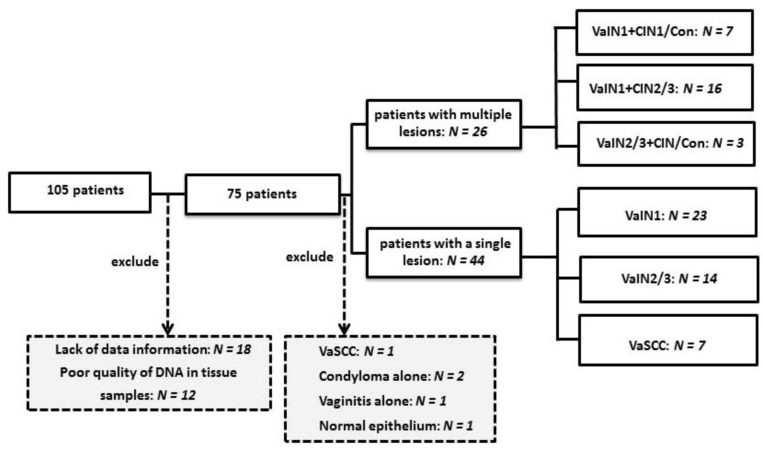
Stratification of patients investigated for vaginal squamous intraepithelial tissue lesions. CIN: cervical intraepithelial neoplasia; Con: condyloma; *N*: number; VaSCC: vaginal squamous cell carcinoma; VaIN: vaginal intraepithelial neoplasia.

In the present study, for convenience the former term, VaIN is used for vaginal squamous intraepithelial lesion (VaSIL), and CIN is used for cervical SIL. One hundred and five patients diagnosed with VaIN and VaSCC, who visited the department of Obstetrics and Gynecology of the Kanazawa Medical University Hospital from 2011 to 2020, were analyzed in this study (Figure 1). All samples were randomized based on the demographic information of all patients. Patients having anal, vulvar, and cervico-vaginal condyloma as the predominant lesion were excluded in this study. All subjects agreed to the use of their cell or histology specimen for this study prior to surgery, according to the protocols approved by the Ethics Committee of Kanazawa Medical University. In total, 12 cases with no or too few histology samples or cell samples and 18 cases with a lack of information were excluded; therefore, 75 patients were evaluated. One pathologist and one surgical pathologist confirmed the diagnosis for each specimen according to the WHO classification [23]. If the diagnosis did not coincide, a final diagnosis was determined via agreement between two pathologists.

Two cases with vaginal condyloma, one with vaginitis, one normal epithelium, and one vaginal cancer case were excluded from further analysis (Figure 1). The origin of this cancer case was not clear because the tumor extended from whole vagina to the cervix. The other five cancer cases presented no tumor in the cervix, and two cases had no uteruses due to surgical resection for fibroids when they were younger. Finally, 70 cases were investigated. Among these, 44 cases had one disease, namely, 23 VaIN1, 14 VaIN2/3, and 7 VaSCC cases. The remaining 26 cases were diagnosed with mixed diseases, namely, 23 cases of VaIN1+CIN/condyloma and 3 cases of VaIN2/3+CIN/condyloma. Cervical (predominantly ex-cervix and cervical canal) scraped cell samples were obtained from all subjects, with the exception of five cases, for HPV genotyping with Genoserach-31+4, as described below. 

### 2.2. HPV Genotyping Using Liquid-Based Cytology (LBC) Specimens with the Genosearch-31+4 Method

LBC samples were obtained from the cervices of most subjects at their first visit to the out-patient clinic in Kanazawa Medical University Hospital. The samples were stored at 4 °C until the HPV genotyping test, which was performed within 2 weeks of the collection by a commercial laboratory (LSI medience, Tokyo, Japan). The HPV genotype was determined using the Genosearch-31+4 (GS-31+4) method (Medical & Biological Laboratories, Co., Ltd., Nagoya, Japan) in the LSI laboratory. This is a new genotyping system based on the PCR-rSSO-Luminex method. This system (GS-31) detects a total of 31 HPV genotypes, including both high-risk (HR), possible high-risk (pHR), and low-risk (LR) HPV types: HPV-6, 11, 16, 18, 26, 31, 33, 35, 39, 42,44, 45, 51, 52, 53, 54, 55, 56, 58, 59, 61, 62, 66, 68,70, 71, 73, 82, 84, 89, and 90. An additional four pHR-HPV types (HPV30, 34, 67, and 69) were also detected with the GS-31+4 system. The GS-31+4 method uses the multiplex primers to amplify different genomic sites of the L1 region on 35 different HPV types, and uses 35 independent probes for detection. It has been confirmed that there is no cross-reaction to other HPV types among these 35 types, and the guaranteed detection limit is 1000 copies of any HPV type in a test [24].

### 2.3. Sandwich Cutting Method for FFPE (Formalin Fixed and Paraffin Embedded) Tissue Specimen Analysis

**Figure 2:**
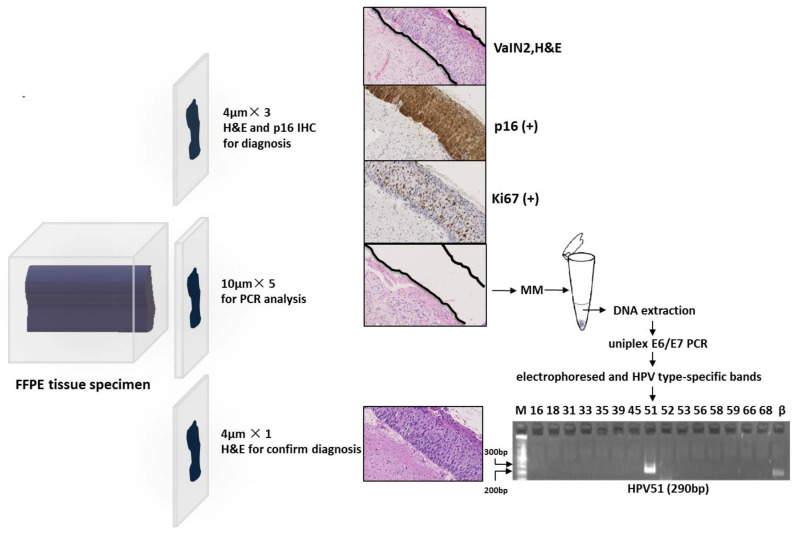
Stratification of patients investigated for vaginal squamous intraepithelial tissue lesions Bp: base pair; FFPE: formalin fixed, paraffin embedded; H&E: haematoxylin and eosin; IHC: immunohistochemistry; M: marker; MM: manual microdissection; PCR: polymerase chain reaction; β: β–globin.

The FFPE blocks for each case were sectioned according to the following procedure: three slices of 4 μM sections were used for histopathological diagnosis after haematoxylin and eosin (H&E) staining, followed by expression of p16 and Ki67 after immunohistochemical (IHC) staining; five slices of 10 μM sections were used for DNA extraction and HPV genotyping; and the final 4 μM section was used for histological evaluation with H&E staining to confirm if the target lesion remained. The microtome was cleaned with 70% alcohol and the blade was replaced for cutting following each tissue block to avoid contamination of HPV in other samples (Figure 2). The tissue slices were placed on individual glass slides that were stored in a refrigerator at −20 °C until DNA extraction.

### 2.4. Immunohistochemical Staining for p16 and Ki67

p16 immunohistochemical staining was performed using the CINtec^®^ Histology Kit (Roche mtm laboratories AG, Heidelberg, Germany) according to the manufacturer’s instructions. Briefly, the 4 µm thick FFPE tissue sections were deparaffinized in xylene and rehydrated in graded alcohols. Antigen retrieval was twice performed in a microwave in Epitope Retrieval Solution (Tris/EDTA, pH 9.0) for 5 min at 500 W. The slides were cooled to room temperature before equilibration in phosphate buffered saline (PBS), and incubated with Peroxidase-Blocking Reagent to block endogenous peroxidase activity. Subsequently, the slides were incubated with the ready-to-use primary anti-p16 antibody (clone E6H4) for 30 min at room temperature. The slides were washed in PBS, then incubated with the secondary antibody (Visualization Reagent) for 30 min at room temperature. The slides were incubated in the Visualization Reagent, which contains a polymer reagent conjugated to horseradish peroxidase (HRP), for 30 min at room temperature. After washing in PBS, incubation with Substrate-Chromogen Solution (1:40 dilution of DAB chromogen in DAB buffer) for 10 min led to p16 brown staining. Mayer’s Hematoxylin was used as a counterstain. Slides were washed in deionized water, dehydrated in graded alcohols, and cleared in xylene. A non-aqueous mounting medium was applied to each section before they were mounted with coverslips. For the detection of the Ki67 antigen, we used the monoclonal mouse antibody (MIB-1, code M7240, Dako, Denmark), and the specific procedure was the same as that described above.

One normal squamous cell tissue without HPV infection was used as the negative control for p16 and Ki67 staining, and two VaIN3 slides with p16 and Ki67 staining were used as the positive control [25]. p16 was expressed in the cytoplasm and/or nucleus in positive cases, whereas Ki67 was expressed only in the nucleus. If more than 5% of squamous cells (nuclear and/or cytoplasmic staining) were positive for p16, staining was considered to be positive for p16 expression. The Ki67 index was defined based on the percentage of positive cells: when the index was below 5% or more than 5%, it was considered to be negative or positive, respectively [25,26,27].

### 2.5. Manual Microdissection and DNA Extraction from Tissue Specimens

All slides stained with H&E (the control slides) were reviewed by a surgical pathologist, and the areas of abnormal lesion, and normal squamous or glandular epithelium, were marked on each slide. By viewing the marked areas of the control slide, an abnormal area or a normal tissue on the tissue slides stained with H&E was independently dissected by hand using a sterilized 21G needle under an inverted microscope (Figure 2). The number of slides used differed in each case according to the size of the target lesion. In the present study, three-to-five slides containing VaIN or CIN were needed to test for 39 HPV types in the uniplex E6/E7 PCR system. Each tissue fragment was placed in a 1.5 mL tube with 50 μL of alkaline lysis reagent (25 mM NaOH, 0.2 mM EDTA, pH 12.0), the tube was incubated in a thermo-shaker (Biosan, TS-100, Latvia) at 95 °C with shaking of 300 revolutions per minute (rpm) for 15 min, and the tube was spun down for 10 s; this procedure was repeated one at 300 rpm and twice at 900 rpm. Then, 50 μL of acidic neutralizing solution (0.04 mM Tris-HCL, pH 5.0) was added, mixed thoroughly, and centrifuged (12,000 rpm) for one minute. A quantity of 50 μL of supernatant was obtained in a new 1.5 mL tube, and diluted five times with distilled water (DW); 5 μL of the solution was used in the uniplex E6-E7 PCR for each HPV type, and the remaining solution was stored in a freezer at −20 °C until the subsequent experiment.

### 2.6. HPV Genotyping Using FFPE Tissue Specimens

The uniplex E6/E7 PCR test was designed to amplify E6 and E7 genes of 39 common HPV types using type-specific primer pairs. This assay is able to individually detect 15 LR-HPV types (HPV6, 11, 40, 42, 44, 54, 55, 61, 62, 71, 74, 81, 84, 89, 90); 11 pHR-HPV types (HPV26, 30, 34, 53, 66, 67, 69, 70, 73, 82, 85); 13 HR-HPV types (HPV16, 18, 31, 33, 35, 39, 45, 51, 52, 56, 58, 59, 68); and the beta-globin gene as a positive control [16].

Using 10 µL of PCR mixture containing 5.0 µL of the sample as a template, 0.5 pM of each HPV type-specific primer, and 5.0 μL of AmpliTaq Gold 360 Master Mix (Applied Biosystem, Foster City, CA), PCR was performed for each HPV type in a T100™ Thermal Cycler (Bio-Rad, Hercules, CA) using the following program: 10 min at 94 °C; 40 cycles of 30 s at 94 °C, 30 s at 60 °C, and 30 s at 72 °C; and a final 5 min extension step. A quantity of 5.0 µL of each reaction solution for each HPV type was applied to a 2.5% agarose gel (Bio-Rad) and electrophoresed in 1× TBE buffer for 8 min. HPV type-specific bands were visualized with SYBR^®^ Green I (Takara-bio, Shiga, Japan) staining in a lane under a UV light (Figure 2).

If the beta-globin gene was negative, it was considered to be a poor quality sample or to have contained an insufficient amount of extracted DNA. These samples were observed in 12 of 30 cases, and were excluded from further analysis (Figure 1).

HPV typing results obtained with the uniplex E6-E7 PCR system were compared with the previous results determined by GS-31+4. The latter HPV test could not detect HPV40, 74, 81, and 85, and among these HPV types, only HPV81 was identified in some samples in the present study.

### 2.7. Statistical Analysis

We used Mann–Whitney’s U test to undertake analysis of continuous variables (patient’s age), and the chi-square test with Yates’ correction was used to compare the categorical variables (prevalence of lesions or HPV-positive cases). We used Fisher’s exact test when the total sample size was less than 10. A probability value of *p* < 0.05 was considered to be statistically significant. All analysis was performed with JSP version 14 (SAS, Tokyo, Japan).

## 3. Results 

### 3.1. Diagnosis and Determination of Vaginal and Cervical Intraepithelial Lesions with P16 and Ki67 Immune-Staining of Tissue Specimens

We used p16 and Ki67 immunohistochemical staining to identify VaIN lesions in tissue specimens using the microdissection procedure. It was shown that 88.2% (15/17) of VaIN2/3 and 28.6% (14/49) of VaIN1 were positive for p16 expression, whereas this was seen in only 9.5% of normal glandular or metaplastic cells, and no expression was found in normal squamous epithelium adjacent to VaIN or CIN. Two VaIN2/3 cases negative for p16 were infected with HPV51 (HR-HPV) and HPV42 (LR-HPV). In contrast, most VaIN1 cases (92.6%, 13/14) positive for p16 expression were infected with HR or pHR-HPV types, although only one p16-positive VaIN1 was infected with an LR-HPV type, namely, HPV71. All VaIN2/3 and 81.6% (40/49) of VaIN1 lesions were positive for Ki67. Of six VaIN1 lesions found to be negative for HPV, four lesions were found to be positive for Ki67 but negative for p16.

### 3.2. The Prevalence of HPV Infection in Cases with Vaginal Intraepithelial Neoplasia (VaIN)

We examined the prevalence of HPV infection in 63 eligible selected cases with VaIN and seven cases with VaSCC. All of these subjects visited our out-patient clinic to undertake further examination for abnormal Pap test worse than atypical squamous cells undetermined significance (ASC-US). All subjects were classified into two groups: the single lesion group, comprising cases with VaIN or VaSCC alone; and the multiple lesions group, comprising cases with VaIN combined with CIN or condyloma. The former group was composed of 23 VaIN1, 14 VaIN2/3, and 7 VaSCC cases, whereas the latter group comprised 7 VaIN1+CIN1 or condyloma, 16 VaIN1+CIN2/3, and 3 VaIN2/3+CIN1 or condyloma (Figure 1). Therefore, 46 cases were diagnosed with VaIN1 and 17 cases with VaIN2/3 (Figure 1).

The comparison of the ages of cases (Table 1) showed no statistical difference between the cases with VaIN1 and those with VaIN2/3, and between cases with a single lesion and those with multiple lesions (Fisher’s test, *p* > 0.1). Seven cases with VaSCC were older (av. 81.7 y) than the other VaIN cases (av. 45.5 y) (Mann–Whitney’s U test, *p* = 0.013).

HPV genotyping in tissue specimens was performed in all the subjects, whereas HPV typing in scraped cell samples was performed in 36 cases with a single VaIN lesion, 24 cases with multiple lesions, and 3 VaSCC cases. Regarding tissue results, overall HPV prevalence in all subjects was 95.7% (Table 1).

Among the single lesion group, the prevalence of any HPV types did not differ between VaIN1 and VaIN2/3 (*p* > 0.1). As for the multiple lesions group, the prevalence of HPV types was calculated separately between VaIN and CIN (Condyloma) groups (Table 2).

### 3.3. Comparison of the Prevalence of HPV Genotypes in Tissue Specimens and in Cervicovaginal Scraped Cells 

HPV genotyping results in the single lesion group are shown in Table 3, and those in multiple lesions are shown in Table 4. First, we analyzed HPV typing results from cervicovaginal cell samples and tissue samples in 39 eligible cases with a single lesion. It was demonstrated that multiple HPV types were detected in 53.8% (21/39) of these cell samples; in contrast, multiple HPV types in tissue samples were identified in only two cases (Table 3). 

When we compared HPV genotype results in tissue and scraped cell samples in the single lesion group (Table 3), eight cases were excluded, because five cases had no HPV data in the scraped cell samples, and three further cases could not be evaluated because HPV81 identified in the tissue specimens was not detectable by another HPV assay (GS-31+4) using the cell sample. This comparison revealed that complete and partial discordance for HPV genotypes between cell and tissue samples was observed in 9 cases (25.0%, 9/36) and 15 cases (41.7%, 15/36), respectively. Therefore, 66.7% (24/36) of these cases showed a discrepancy in the HPV genotype between cell and tissue samples. Moreover, 15 cases (41.7%, 15/36) that were positive for certain HR-HPV types detected in cell samples were not present in the tissue specimen (Table 3). About 40% of these preclinical HR-HPV infections were observed in cases with a single VaIN. This was more common in the case of VaIN1 (11/19, 57.9%) than in VaIN2/3 (2/14, 14.3%) (*p* = 0.030).

The same analysis was performed for 24 eligible cases with multiple lesions who undertook HPV testing using both cell and tissue samples. Various HPV types that had been detected in cell samples were not present in 70.8% (17/24) of tissue specimens. Regarding HR-HPV type, these preclinical infections were recognized in 54.2% (13/24) of the multiple lesion group. Conversely, the opposite result, in which the HPV genotype was not identified in cell samples but was positive in tissues, was seen in two cases (Cases #34, 48). 

It is surprising that the frequency of the preclinical HPV infection did not differ between the single lesion and multiple lesion groups (Fisher’s test, *p* > 0.1). In all subjects, 68.3% (41/60) had preclinical infection with any HPV type, and 46.7% (28/60) were found to have a preclinical HR-HPV-type infection. Moreover, HPV genotypes in VaIN and CIN lesions differed in 92.3% (24/26) of cases with multiple lesions (Table 4). This suggests that the vaginal and cervical lesions developed independently in most cases. 

### 3.4. Determination of HPV Genotypes Responsible for VaSCC, VaIN, and CIN by Molecular Mapping

Epithelial tissues including target lesions were manually dissected for HPV genotyping in all cases, and DNA samples were obtained from 99 sections and evaluated for HPV genotypes. It was revealed that 57 of 63 (90.5%) VaIN tissues were positive for HPV (Table 3 and Table 4). One VaIN tissue in the single lesion group, and five VaIN tissues in the multiple lesion group, were found to be negative for any HPV type (Table 3 and Table 4). Two cases in the single lesion group (Cases #19, 23) and four cases of the multiple lesion group (Cases #32, 46, 48, 63) showed multiple HPV type infections in one tissue specimen (Table 3 and Table 4). We re-evaluated the histology of these six specimens that showed multiple HPV type infections, and found that morphologically different areas existed in some slides for each case. Two or three separable lesions were cut out by manual dissection under the guidance of counter-staining with hematoxylin and expression of p16 or Ki67, as described in the Materials and Methods section (Figure 2). This revealed that different HPV types were identified in each of VaIN2/3 and VaIN1 lesions in Case#19, Case #23, Case #32; VaIN1 and metaplasia in Case #46; CIN2/3 and CIN1 lesions in Case#63; and two CIN1 lesions in Case #48 (Table 3 and Table 4). 

In 49 VaIN1 lesions, 17 HPV types were detected in 43 lesions (87.8%, 43/49), including HR-HPV in 6 lesions, pHR-HPV in 18 lesions, and LR-HPV in 19 lesions. The most common type was HPV53, which was found in six VaIN1 lesions, followed by HPV66/81/90 in five lesions, HPV34/67/71 in three lesions, HPV42/61/84 in two lesions, and HPV16/31/52/56/58/68/73 in one lesion. In higher-grade lesions, eight HPV types were identified in VaIN2/3, whereas six HPV types were found in VaSCC. The most common type in VaIN2/3 was HPV16 (HR-HPV) (35.3%, 6/17), followed by HPV51/52/58 (HR-HPV) (11.8%, 2/17), HPV42 (LR-HPV) (11.8%, 2/17), and HPV18/56 (HR-HPV) and HPV66 (pHR-HPV) in one case each. In contrast, HPV16/45/58/68 (HR-HPV) and HPV53/67 (pHR-HPV) were identified as one HPV type in each case of VaSCC, and a negative result was detected in one case. Among the 13 internationally defined HR-HPV types, four HPV types (HPV33, 35, 39 and 59) were not detected in any VaIN lesions in the present study, although HPV39 was identified in two CIN2 cases (Table 4 and Table 5).

The prevalence of HR-HPV types was more common in VaIN2/3 (85.7%) than in VaIN1 (17.4%) (*p* < 0.001), whereas possible HR (pHR) HPV types were more common in VaIN1 (34.8%) than VaIN2/3 (5.9%) (*p* = 0.015). The LR-HPV type was marginally more prevalent in VaIN1 than VaIN2/3 (*p* = 0.084) (Table 5).

## 4. Discussion

The present study showed the prevalence of HPV was 95.7% in low-grade vaginal squamous intraepithelial lesions (VaIN1s), 100% in high-grade lesions (VaIN2 and VaIN3), and 85.7% in cancer (VaSCC). Different studies have shown different prevalence results of HPV in VaINs. A meta-analysis showed that the overall prevalence of HPV in VaIN (*n* = 1374) was 85.2%, with the prevalence of each study ranging from 20% to 100% [28]. Frega et al. [29] reported that the HPV positive rate in VaIN cases is 100%; however, 40 of 44 of these cases had a past history of cervical carcinoma. In contrast, Tsimplaki et al. [30] reported that the prevalence of HPV was 56% in VaIN cases who had no history of other HPV-associated malignancies. This difference is likely to be due to selection bias or different HPV detection methods. The former report selected a higher risk group because most cases have a past history of cervical cancer. To examine HPV genotypes in VaIN cases, this report examined scraped cell samples, and found that all samples were positive for HPV16 (80%) or HPV18 (20%). Using whole FFPE tissue samples of VaIN, Tsimplaki et al. [30] identified 24 HPV types, including 13 HR-HPV types, 5 pHR-HPV types, and 6 LR-HPV types. Chao et al. [31] investigated 450 VaIN cases for HPV genotyping using FFPE whole tissue specimens, and testing was able to detect 38 HPV types. This report showed that 41.8% of VaIN1 and 58.2% of VaIN2/3 were positive for HPV. Although this study was similar to the present study regarding the sample and HPV detection procedure, the present study showed a higher prevalence of HPV, which was more than 96%. This difference might be due to the difference in the method used. The studies of Tsimplaki et al. and Chao et al. used whole FFPE tissue specimens, whereas the present study used an epithelial tissue of the target lesion for HPV genotyping. Quint W et al. [32] already demonstrated that 100% of anal intraepithelial neoplasia (AIN) samples, which were obtained via laser capture microdissection, were positive for HPV using the SPF10-PCR method. The present study may be the first to demonstrate that the manual microdissection of VaIN or CIN epithelia from the FFPE tissue specimen is as effective as the LCM procedure in the other studies [20]. In our preliminary experiment, HPV and the beta-globin gene, as the internal control, were not detected in some of the cervical cancer specimens that were taken more than 10 years ago. Furthermore, in addition to the quality of the sample, molecular mapping of the target lesion is also crucial for successful HPV genotyping analysis of a fragment as small as that of VaIN. Therefore, the difference in HPV prevalence in VaIN reported in different studies may be due to sample selection, and the size and quality of the tissue sample. 

In the present study, 63 VaIN cases were diagnosed randomly in women who were referred to our out-patient clinic due to an abnormal Pap test result or for follow up for CIN1, 2. This study used a highly sensitive HPV test, uniplex E6/E7 PCR, which is able to detect 39 HPV types, including all HR- and pHR-HPV types, and some LR-HPV types. As mentioned above, all VaIN2/3 lesions were positive for HPV, suggesting that the uniplex E6/E7 PCR method may encompass almost all of the HPV genotypes that might be responsible for VaIN2/3. 

Histological evaluation demonstrated 37 cases (56.6%) with VaIN alone, and 26 (40.0%) cases with VaIN and CIN or condyloma. In the latter cases, 24 cases (92.3%) were infected with different HPV types in each lesion of VaIN and CIN; this result was not expected. This suggests that VaIN and CIN were independently developed. Moreover, HPV genotyping results from the cell samples showed additional HPV types that were not identified. Okodo M et al. [21] confirmed that HPV types determined using the uniplex E6/E7 PCR method, which was used in the present study, were highly consistent with the results achieved from the GS-31+4 test using the same cell samples. Therefore, the discrepancy in the HPV results between different samples was not due to the difference in the assay used. The present analysis using the microdissection procedure clearly demonstrated a single HPV type in one lesion. The two assays used in the present study theoretically provide results that are specific to the HPV type. Therefore, the above-mentioned additional HPV types that were detected only in cell samples may be derived from preclinical infections somewhere in the cervix or vagina. If this hypothesis is correct, these preclinical infections existed in 69% of VaIN cases, and those with HR-HPV types were seen in 45% of cases. Van Der Marel J et al. [19] also reported that, based on cytology samples, 62% of the CIN3 women had infections of multiple HPV types, whereas the cytological HPV genotypes were not detected in the corresponding CIN3 lesions. These findings indicate that HPV genotype results determined from scraped cell samples are not always derived from a target lesion defined by colposcopy. For this reason, only a single HPV type result was adopted as the type responsible for the lesion in our previous studies [16,18].

In patients with multiple lesions, 92.3% (24/26) had completely different HPV types in the lesions of CIN and VaIN (Table 4). In these cases, 12 cases had different grades of lesions in different areas; VaIN2/3+CIN1 or CIN2/3+VaIN1, and HR-HPV, were found in all high-grade lesions, and LR- or pHR-HPV were found in seven low-grade lesions in different sites, such as VaIN1+CIN1 or VaIN1+congdyloma. This can also indicate that high-grade lesions are caused by HR-HPV types, whereas low-grade lesions are caused by low-risk or pHR-HPV types. This tendency was statistically relevant. High-grade lesions, including 17 VaIN2/3 and 7 VaSCC tissue specimens, were infected with HR HPV types—HPV16, 52, 58 (Alpha-9), 18, 45 (Alpha-7), 51 (Alpha-5), and 56 (Alpha-6); two lesions with LR-HPV type 42 (Alpha-1); and one lesion with pHR-HPV type 53, 66 (Alpha-6), and 67 (Alpha-9). Okodo M et al. [21] reported that no LR-HPV types were identified as a single-type infection in CIN2/3 and cancer; this is consistent with the present results, in which no low-risk types were identified in 14 specimens of CIN2/3. Similarly, 14 VaIN2/3 and 4 VaSCC lesions were positive for HR-HPV, 1 VaIN2/3 and 2 VaSCC for pHR-HPV (HPV53, 66, 67), and 2 VaIN2/3 for HPV42. In our previous study, HPV52, 16, 51, 56, and 42 were identified as a single type in VaIN2/3 [16]. Summarizing both sets of data, the most common species was Alpha-9 (HPV16, 52, 58, 67) (13 lesions), followed by Alpha-6 (HPV53, 56, 66) (3 lesions), Alpha-7 (HPV18, 45, 68) (3 lesions), Alpha-5 (HPV51) (2 lesions), and Alpha-1 (HPV42) (2 lesions). These results suggest that almost all were HR- or pHR-HPV types. Some studies suggest that some pHR-HPV types, such as HPV26, 66, 67, 69, 73, and 82, are likely to be oncogenic [8,9,10] More surprisingly, one LR-HPV type (HPV42) (Alpha-1) was detected in two cases of VaIN2/3. Guimera et al. [33] demonstrated that HPV42 is found in a typical squamous cell carcinoma of the cervix. Further study is needed to confirm the oncogenicity of this type.

We used p16 and Ki67 immunohistochemical staining to identify VaIN lesions in tissue specimens using the microdissection procedure. It was shown that most of VaIN2/3 (15/17) and 28.6% (14/49) of VaIN1 were positive for p16 expression, and no expression was found in normal squamous epithelium adjacent to VaIN or CIN. Two VaIN2/3 lesions negative for p16 were infected with HPV51 (HR-HPV) and HPV42 (LR-HPV), whereas the other cases that were positive for p16 were infected with HR or pHR HPV types. Moreover, most VaIN1 cases (92.6%; 13/14) positive for p16 expression were infected with HR- or pHR-HPV types, although only one p16-positive VaIN1 was infected with HPV71 (LR-HPV). In contrast, all VaIN2/3 and most VaIN1 (81.6%; 40/49) were positive for Ki67. Despite some exceptions, Ki67 expression is useful to discriminate any VaIN lesions from normal squamous epithelium, and p16 expression is associated with HR-HPV or pHR-HPV infection, regardless of the grades of vaginal lesion. Similar conclusions were reported by Chao et al. [31]. In the present study, among the six VaIN1 lesions that were found to be negative for HPV, four HPV-negative lesions were positive for Ki67 expression, but negative for p16, suggesting that these lesions might be infected with LR-HPV types that are unable to be detected by the present HPV test. Low viral load and DNA fragmentation are likely to be the cause of these negative results. 

The present study showed that HR-HPV is more common in VaIN2/3, whereas pHR- and LR-HPV are more common in VaIN1. VaIN1 were positive for any type of HPV. Castle et al. reported that LR-HPV types belonging to Alpha-3 and -15 are more prevalent in the vagina than in the cervix [16]. Most LR-HPV types in the present study belonged to Alpha-3 (HPV61, 81, 84) and Alpha-15 (HPV71, 90). These findings may support the conclusion that the vagina generally functions as a reservoir for many mucosal HPV types, and only HR- or pHR-HPV types can induce cancer if persistent infections with these types are established.

The ages of VaSCC patients were greater than those of VaIN cases in the present study (the average age of VaSCC cases was 81.7 years, which was greater than that of VaIN cases), suggesting long latency is needed for malignant progression. It was noted that six different HR- and pHR-HPV types were identified in each of the VaSCC cases, suggesting that various HPV types are responsible for VaSCC development. In particular, two of the six (33.3%) cancers were infected with pHR types (HPV53, 67), suggesting that the prevalence of pHR types appears to be greater in VaSCC than in CxSCC [18]. Sakamoto et al. determined HPV type in CxSCC cases with the same method as this paper, the results revealed that he prevalence of HPV16 and 18 are lower in VaSCC than that in CxSCC (*p* = 0.019), although further study is needed to warrant it.

The present study also demonstrated that the manual microdissection procedure, which is a cost-effective method, is as effective as LCM for the acquisition of target tissue for HPV genotyping [20]. From a clinical perspective, the present study suggests that high-grade VaIN could be detected with a highly sensitive HPV test in cervical cancer screening and careful examination by colposcopy. Clinicians should also note that some VaIN lesions may be found in women who show false positive results in their cervix during screening. To reduce the incidence of cervical cancer in many countries, the nonavalent HPV vaccine, which protects against HPV6, 11, 16, 18, 31, 33, 45, 52, and 58 infections, has recently been launched [34]. The present study suggests that the nonavalent HPV vaccine could protect against 64.7% of VaIN2/3 and 42.9% of VaSCC in Japan. Similarly, the bivalent or quadrivalent HPV vaccines could protect against 41.2% of VaIN2/3 and 14.3% of VaSCC. 

This study has some limitations. The most important of these is the small size of the samples of VaIN and, in particular, VaSCC, which comprised only seven cases. Thus, similar studies are needed to increase the sample size for further research.

## 5. Conclusions

Many HPV types, including high-risk (HR), possible high-risk (pHR), and low-risk (LR) types, were identified in low-grade vaginal intraepithelial lesion (VaIN1), whereas HR and pHR-HPV types were identified in high-grade vaginal lesions (VaIN2/3). The vagina appears to be the reservoir for any mucosal HPV type, and HR or pHR HPV types are causative agents for vaginal malignancies. Different HPV type distribution between the vagina and the cervix suggests that CIN and VaIN are independently developed in most cases.

## Figures and Tables

**Table 1:** Age of patients, histological diagnosis and HPV infection by E6/E7 PCR using tissue specimens.

	Cases with a Single Lesion			Cases with Multiple Lesions					
Diagnosis	VaIN1	VaIN2/3		VaSCC		VaIN1+CIN1/Con	VaIN1+CIN2/3	VaIN2/3+CIN/Con	Total
	*N* = 23	*N* = 14	*p*¹	*N* = 7	*p* ^2^	*N* = 7	*N* = 16	*N* = 3	*N* = 70
**Age**	43.8 (27–67)	48.3 (22–55)	*>0.1*	81.7 (76–88)	*0.013*	36.6 (19–55)	37.8 (22–80)	42.4 (32–50)	48.6 (19–88)
**HPV prevalence (%)**	95.7 (22/23)	100 (14/14)	*>0.1*	85.7 (6/7)		85.6 (6/7)	100 (16/16)	100 (3/3)	95.7 (67/70)
HR-HPV (%) *	17.4 (4/23)	85.7 (12/14)		57.1 (4/17)		28.6 (2/7)	93.8 (15/16)	66.7 (2/3)	55.7 (39/70)
Possible HR-HPV (%) *	34.8 (8/23)	0 (0/14)		28.6 (2/7)		57.1 (4/7)	43.8 (7/16)	33.3 (1/3)	31.4 (20/70)
LR-HPV (%) *	43.5 (10/23)	14.3 (2/14)		0 (0/7)		42.9 (3/7)	25.0 (4/16)	66.7 (2/3)	30.0 (21/70)
**Single HPV type (%)**	95.7 (22/23)	78.6 (11/14)		85.7 (6/7)		14.3 (1/7)	25.0 (4/16)	0 (0/3)	62.9 (38/70)
**Multiple HPV types (%)**	0 (0/23)	21.4 (3/14)		0 (0/7)		71.4 (5/7)	75.0 (12/16)	100 (3/3)	32.9 (23/70)

*: Numbers of HPV types identified as one of the multiple HPV types are repeatedly counted. CIN, cervical intraepithelial neoplasia; Con, condyloma; HPV, Human papillomavirus; HR-HPV, High risk-HPV; LR-HPV, Low risk-HPV; N, number; VaSCC, vaginal squamous cell carcinoma; VaIN, vaginal intraepithelial neoplasia. *p*¹ value means comparing the age, and the prevalence of any HPV type between VaIN1 and VaIN2/3 cases. *p*^2^ value means comparing the age between all VaIN and VaSCC cases.

**Table 2:** Age of patients, histological diagnosis and HPV infection by E6/E7 PCR using tissue specimens.

	Cases with a Single Lesion			Cases with Multiple Lesions					
Diagnosis	VaIN1	VaIN2/3		VaSCC		VaIN1+CIN1/Con	VaIN1+CIN2/3	VaIN2/3+CIN/Con	Total
	*N* = 23	*N* = 14	*p*¹	*N* = 7	*p* ^2^	*N* = 7	*N* = 16	*N* = 3	*N* = 70
**Age**	43.8 (27–67)	48.3 (22–55)	*>0.1*	81.7 (76–88)	*0.013*	36.6 (19–55)	37.8 (22–80)	42.4 (32–50)	48.6 (19–88)
**HPV prevalence (%)**	95.7 (22/23)	100 (14/14)	*>0.1*	85.7 (6/7)		85.6 (6/7)	100 (16/16)	100 (3/3)	95.7 (67/70)
HR-HPV (%) *	17.4 (4/23)	85.7 (12/14)		57.1 (4/17)		28.6 (2/7)	93.8 (15/16)	66.7 (2/3)	55.7 (39/70)
						VaIN1 CIN1/Con	VaIN1 CIN2/3	VaIN2/3 CIN/Con	
						0 (0/7) 28.6 (2/7)	6.25 (1/16) 87.5 (14/16)	66.7 (2/3) 33.3 (1/3)	
Possible HR-HPV (%) *	34.8 (8/23)	0 (0/14)		28.6 (2/7)		57.1 (4/7)	43.8 (7/16)	33.3 (1/3)	31.4 (20/70)
						VaIN1 CIN1/Con	VaIN1 CIN2/3	VaIN2/3 CIN/Con	
						42.9 (3/7) 28.6 (2/7)	43.8 (7/16) 12.5 (2/16)	33.3 (1/3) 33.3 (1/3)	
LR-HPV (%) *	43.5 (10/23)	14.3 (2/14)		0 (0/7)		42.9 (3/7)	25.0 (4/16)	66.7 (2/3)	30.0(21/70)
						VaIN1 CIN1/Con	VaIN1 CIN2/3	VaIN2/3 CIN/Con	
						42.9 (3/7) 28.6 (2/7)	25.0 (4/16) 0 (0/16)	33.3 (1/3) 33.3 (1/3)	
**Single HPV type (%)**	95.7 (22/23)	78.6 (11/14)		85.7 (6/7)		14.3 (1/7)	25.0 (4/16)	0 (0/3)	62.9(38/70)
**Multiple HPV types (%)**	0 (0/23)	21.4 (3/14)		0 (0/7)		71.4 (5/7)	75.0 (12/16)	100 (3/3)	32.9(23/70)

*: Numbers of HPV types identified as one of the multiple HPV types are repeatedly counted. CIN, cervical intraepithelial neoplasia; Con, condyloma; HPV, Human papillomavirus; HR-HPV, High risk-HPV; LR-HPV, Low risk-HPV; N, number; VaSCC, vaginal squamous cell carcinoma; VaIN, vaginal intraepithelial neoplasia. *p*¹ value means comparing the age, and the prevalence of any HPV type between VaIN1 and VaIN2/3 cases. *p*^2^ value means comparing the age between all VaIN and VaSCC cases.

**Table 3:** Comparison of HPV type results determined by tissue or scraped cell samples in cases with a single lesion.

Case	VaIN	HPV Type in Tissue	HPV Type in Scraped	Discordance in HPV Type	HR-HPV May Exist
No.		by Uniplex E6/E7 PCR	Cells by GS31+4	in Tissue and Cell Samples	in Preclinical Lesions
1	VaIN1	HPV81 *	Negative	NE	
2	VaIN1	HPV81 *	HPV56,58	NE	56, 58
4	VaIN1	HPV84	HPV51,58	Completely	51,58
7	VaIN1	HPV90	HPV16,90	Partly	16
8	VaIN1	HPV81 *	HPV51,66,89,42	NE	51
11	VaIN1	HPV66	HPV66,89	Partly	
16	VaIN1	Negative	HPV31	Completely	31
18	VaIN1	HPV16	HPV31,82	Completely	31
27	VaIN1	HPV53	HPV53	No	
28	VaIN1	HPV90	HPV55,61	Completely	
29	VaIN1	HPV53	HPV53,62	Partly	
30	VaIN1	HPV68	HPV52	Completely	52
33	VaIN1	HPV66	HPV18,51,61,66,84,90	Partly	18, 51
36	VaIN1	HPV66	HPV66	No	
39	VaIN1	HPV61	HPV39,61	Partly	39
40	VaIN1	HPV61	HPV31,61	Partly	31
45	VaIN1	HPV58	HPV58	No	
50	VaIN1	HPV67	HPV67	No	
52	VaIN1	HPV73	HPV73	No	
53	VaIN1	HPV90	Negative	Completely	
55	VaIN1	HPV56	HPV52,56	Partly	52
59	VaIN1	HPV71	Negative	Completely	
62	VaIN1	HPV34	NE	NE	
5	VaIN2/3	HPV52	HPV52	No	
12	VaIN2/3	HPV18	HPV18	No	
14	VaIN2/3	HPV16	HPV16,84,89,90	Partly	
17	VaIN2/3	HPV52	HPV52,84	Partly	
25	VaIN2/3	HPV42	HPV6,42	Partly	
57	VaIN2/3	HPV42	HPV42	No	
6	VaIN2/3	HPV16	HPV16,89	Partly	
19	VaIN2/3+VaIN1	HPV58+HPV31	HPV31,54,58,90	Partly	
23	VaIN2/3+VaIN1	HPV51+HPV90	HPV39,51,90	Partly	39
41	VaIN2/3	HPV16	HPV16	No	
42	VaIN2/3	HPV16	HPV68	Completely	
44	VaIN2/3	HPV56	HPV56	No	
51	VaIN2/3	HPV16	HPV16	No	
54	VaIN2/3	HPV58	HPV39,58	Partly	39
64	VaSCC	HPV16	HPV16	No	
65	VaSCC	HPV45	NE	NE	
66	VaSCC	HPV58	HPV16,52,58	Partly	16,52
67	VaSCC	HPV68	NE	NE	
68	VaSCC	HPV53	NE	NE	
69	VaSCC	HPV67	HPV6,52	Completely	52
70	VaSCC	Negative	NE	NE	

HPV, Human papillomavirus; HR-HPV, high-risk HPV; No, number; VaIN, vaginal intraepithelial neoplasia; VaSCC, vaginal squamous cell carcinoma; NE, not examined or not evaluated for undetectable HPV type by GS31+4; *, HPV81 is not detectable by GS31+4; *p* value means comparing preclinical HR-HPV infection between VaIN1 and VaIN2/3 cases.

**Table 4:** Compariosn of HPV type results determined by tissue or scraped cell-samples in cases with multiple lesions.

VaIN	HPV Type in Tissue	Acompanied	HPV Type in Tissue	Discordance for HPV Type	HPV Type in Cells	Discordance for HPV Type	HR-HPV May Exist
Lesion	by E6/E7 PCR	Lesion	by E6/E7 PCR	VAIN vs. CIN/Con	by GS-31+4	Tissue vs. Cells	in Preclinical Lesions
VaIN1	HPV53	CIN3	HPV52	Yes	HPV16,39,52,90	Partly	16,39
VaIN1	Negative	CIN2	HPV18	Yes	HPV18	No	
VaIN1	Negative	CIN2	HPV52	Yes	HPV16,52	Partly	16
VaIN1	HPV81	CIN1	HPV90	Yes	HPV51,52,58,59,90	Partly	51,52,58,59
VaIN1	Negative	CIN1	Negative	No	Negative	No	
VaIN1	Negative	CIN3	HPV51	Yes	HPV51	No	
VaIN1	HPV42	CIN2	HPV52	Yes	HPV42,52	No	
VaIN1	HPV53	CIN1	HPV68	Yes	HPV52,53,68	Partly	52
VaIN1	HPV42	CIN2	HPV58	Yes	HPV31, 51	Completely	31, 51
VaIN1	HPV67	CIN2	HPV52	Yes	HPV52	Partly	
VaIN1	HPV67	CIN1	HPV67	No	Negative	Completely	
VaIN1	HPV81	CIN3	HPV16	Yes	HPV16,35,51,53	NE	35,51
VaIN1	HPV71	CIN1	HPV84	Yes	HPV11,16,53,54,62,84	Partly	16
VaIN1	HPV52	CIN2	HPV66	Yes	HPV16,52,66	Partly	16
VaIN1+metaplasia	HPV66+HPV74	CIN1	HPV53	Yes	HPV42,52,53	Partly	52
VaIN1	HPV53	Condyloma	HPV11	Yes	HPV56,53,73	Partly	56
VaIN1	HPV90	CIN1+CIN1	HPV52+HPV68	Yes	HPV52,68	Partly	
VaIN1	HPV34	CIN2	HPV39	Yes	NE	NE	
VaIN1	HPV34	CIN3	HPV52	Yes	HPV34,52	No	
VaIN1	HPV53	CIN2	HPV39	Yes	HPV16,42,89	Completely	16
VaIN1	HPV84	CIN3	HPV52	Yes	NE	NE	
VaIN1	Negative	CIN2	HPV51	Yes	HPV51	No	
VaIN1	HPV66	CIN3+CIN1	HPV16+HPV66	Yes	HPV16,52,59,53,66,82	Partly	52,59
VaIN2/3	HPV66	CIN1	HPV67	Yes	HPV82	Completely	
VaIN2/3	HPV16	Condyloma	HPV6	Yes	HPV6,16	No	
VaIN2/3+VaIN1	HPV51+HPV71	CIN2	HPV51	Yes	HPV51,71,68	Partly	68

CIN, cervical intraepithelial neoplasia; Con, condyloma; HPV, Human papillomavirus; HR-HPV, High risk-HPV; LR-HPV, Low risk-HPV; N, number; VaIN, vaginal intraepithelial neoplasia; NE, Not examined or Not evaluated for undetectable HPV type by GS31+4.

**Table 5:** Identified HPV type in a histological tissue of VaIN, VaSCC and CIN.

	HPV Type	VaIN1	VaIN2/3		VaSCC	Total	CIN1	CIN2/3	Total
		*N* = 49 *	*N* = 17	*p*	*N* = 7	No.	*N* = 10	*N* = 16	No.
**High-risk HPV**	**HPV16**	1	6		1	8		2	2
	**HPV18**		1			1		1	1
	**HPV31**	1				1			
	**HPV39**							2	2
	**HPV45**				1	1			
	**HPV51**		2			2		3	3
	**HPV52**	1	2			3	1	6	7
	**HPV56**	1	1			2			
	**HPV58**	1	2		1	4		1	1
	**HPV68**	1			1	2	2		2
**Possibly high-risk HPV**	**HPV34**	3				3			
	**HPV53**	6			1	7	1		1
	**HPV66**	5	1			6	1	1	2
	**HPV67**	3			1	4	2		2
	**HPV73**	1				1			
**Low-risk HPV**	**HPV42**	2	2			4			
	**HPV61**	2				2			
	**HPV71**	3				3			
	**HPV81**	5				5			
	**HPV84**	2				2	1		1
	**HPV90**	5				5	1		1
	**Negative**	6	0		1	7	1	0	1
**Any HPV type**		43 (87.8%)	17 (100%)		6 (85.7%)	66 (90.4%)	9 (90.0%)	16 (100%)	25 (96.1%)
**HR-HPV type**		6	14	<0.001	4	24	3	15	18
**Possibly HR-HPV type**		18	1	0.015	2	21	4	1	5
**LR-HPV type**		19	2	0.084	0	21	2	0	2
**Total**		49	17		7	73	10	16	26

*: *N* means the number of the tissue specimens, CIN, cervical intraepithelial neoplasia; HPV, Human papillomavirus; HR-HPV, High risk-HPV; LR-HPV, Low risk-HPV; N, number; VaSCC, vaginal squamous cell carcinoma; VaIN, vaginal intraepithelial neoplasia. *p* value means comparing the prevalence of HPV infection between VaIN1 and VaIN2/3 cases.

## Data Availability

This study did not report any data.

## Abbreviations

HPV	human papillomavirus
Vag-SIL	vaginal squamous intraepithelial lesion
VaIN	vaginal intraepithelial neoplasia
VaSCC	vaginal squamous cell carcinomas
CIN	cervical intraepithelial neoplasia
GS-31+4	Genosearch-31+4
HR	high risk
pHR	possibly high risk
LR	low risk
dsDNA	double-stranded DNA
pRb	retinoblastoma protein
SCJ	squamous-columnar junction
LCM	laser capture microdissection
FFPE	formalin-fixed and paraffin-embedded
PCR	polymerase chain reaction
H&E	hematoxylin and eosin
IHC	immunohistochemistry
PBS	phosphate buffered saline
HRP	horseradish peroxidase
DAB	3,3′-diaminobenzidine
DW	distilled water
